# Low-power NIR-mediated photothermal and photodynamic therapy as a synergistic strategy against superbugs

**DOI:** 10.3389/fmicb.2026.1877322

**Published:** 2026-07-28

**Authors:** Tapan Kumar Panda, Irfhan Sheriff, Jyoti Patel G, Krishna Rao Eswar Neerugatti

**Affiliations:** 1 Centre for Nanobiotechnology (CNBT), Vellore Institute of Technology, Vellore, India; 2 School of Bio Science and Technology, Vellore Institute of Technology, Vellore, India

**Keywords:** antibacterial, low power NIR, photodynamic therapy, photothermal therapy, superbugs

## Abstract

Antibiotic resistance is overburdening the health crisis around the globe. Misuse or overuse of antibiotics is one of the reasons for this issue. Therefore, it urges an alternative treatment strategy that can overcome the issue of bacteria being resistant. Photothermal and photodynamic therapies are emerging strategies, being an antibiotic-free solution to treat infections. This method utilizes antimicrobial treatment with heat and ROS, which makes it challenging for bacteria to become resistant. Earlier photothermal therapies required high-power laser sources, which had a significant risk of damaging healthy tissues in the vicinity. Recent research has shown that low-power NIR sources have great potential to achieve equivalent photothermal conversion efficiency when used with different sets of engineered photothermal agents and are also safe for use. A synergistic, low-power antimicrobial strategy combining the photodynamic properties and photothermal properties of the materials results in better efficiency. Additionally, leveraging the NIR-II range allows for deeper penetration into tissues, making it effective to treat deep-seated infections. This review highlights recent advancements in low-power NIR mediated PTT/PDT for mitigating drug-resistant bacteria, focusing on mechanisms, therapeutic outcomes, challenges, and future prospects.

## 1 Introduction

Infectious diseases caused by bacteria are a top public health threat around the world (Zhao et al., 2020; Zhou K. et al., 2020). Antibiotic resistance developed in bacteria has amplified this issue (Liu et al., 2021). It is a rapidly growing global health crisis. Recent data shows that 1 in 6 bacterial infections are now resistant to standard antibiotics, with resistance rates rising by 5%–15% annually. WHO projects that AMR could be directly responsible for 39 million deaths between 2025 and 2050 (Endale et al., 2023; World Health Organization [WHO], n.d.). The accelerated emergence of bacterial adaptation and resistance is due to the misuse and overuse of antibiotics for several decades (Liu et al., 2021). The emergence of bacterial biofilms is a significant consequence of increasing resistance (Zhao et al., 2020). Bacteria inside biofilms show more resistance to antibiotics and the host’s defense systems compared to planktonic bacteria. This resistance renders antibiotic therapy nearly impossible (Zhang et al., 2019).

Due to the limitations of antibiotic development and the need for novel antibacterial means of treatment, researchers are investigating alternative methods of treatments that are targeted, minimally invasive, and do not result in the resistance development (Zhou K. et al., 2020). Specifically, light-based treatments, namely photothermal therapy (PTT) and photodynamic therapy (PDT), are promising non-antibiotic methods in the fight against superbugs (Zhang et al., 2021). Both of these approaches use light with intrinsic benefits of non-invasiveness, temporal-spatial control, and inability to develop drug resistance (Zhang et al., 2018).

Photosensitizers (PSs) are employed in PDT because they can generate ROS, like singlet oxygen, in the presence of light energy in the NIR spectrum (Zhang et al., 2018). The ROS produced will damage bacterial proteins, nucleic acids, lipids, and outer structures (Wang et al., 2022). Bacteria have a difficult time becoming resistant to PDT because it employs a treatment algorithm on multiple targets (Wang et al., 2022). PTT uses photothermal agents (PTAs), which are effective in generating localized heat from absorbed light energy, especially in the near-infrared region (Hou et al., 2022). Thermoablation eliminates bacteria physically by causing protein denaturation and rupture of the cell membrane by elevating the local temperature to levels (typically >50 °C) (Zhang et al., 2019). It’s believed that PTT is a promising non-resistant antibacterial treatment because bacteria generally have a poor capability to withstand heat (Zhang et al., 2019; Wang et al., 2022). Combination of both PTT and PDT is found to be more effective while using a single PS and NIR light source. However, there is a limitation of high-power NIR sources for PTT and PDT which is causing harm to the nearby tissues at the point of application. Several reviews have discussed about photothermal antibacterial mechanisms (Qi et al., 2023; Gong et al., 2025; Zhang and Li, 2026) but this review highlights the limitation of conventional methods and emphasizing on low power (≤1 W/cm^2^) NIR based PTT and PDT as it is vital in the perspective of translation to the real life applications.

## 2 Limitation of conventional PTT and PDT

**FIGURE 1:**
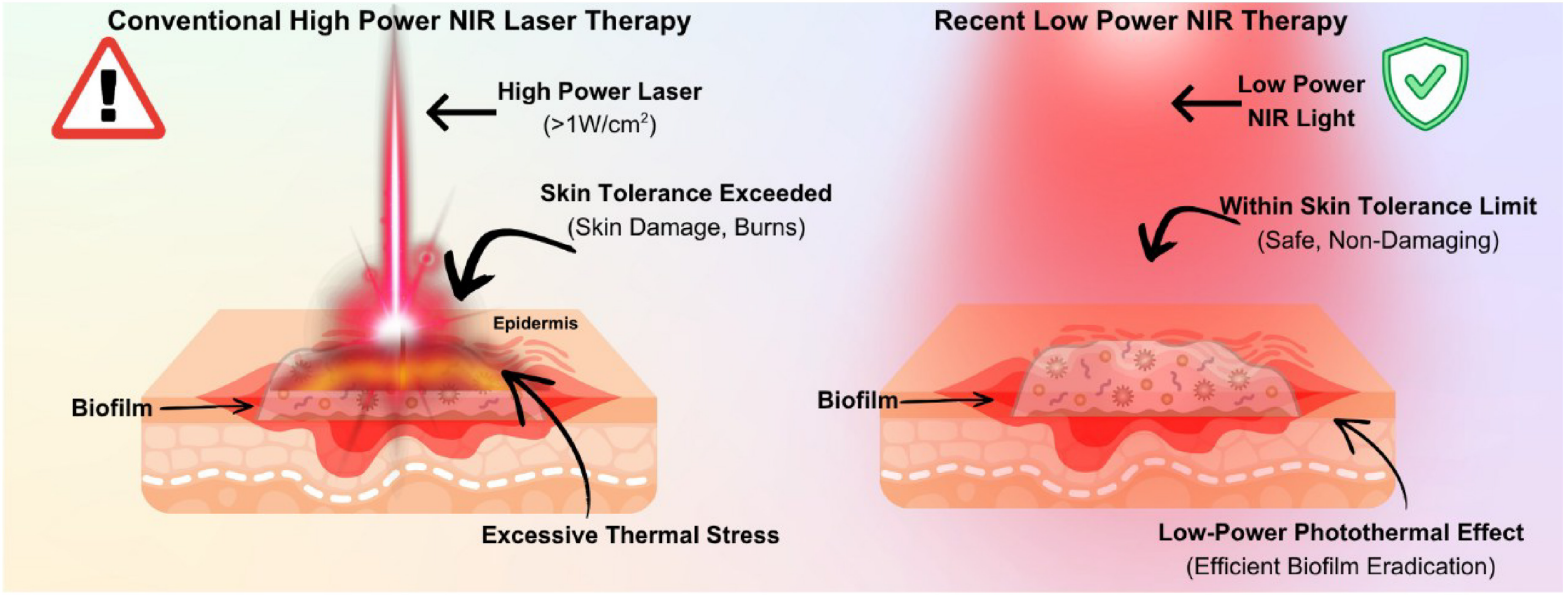
Effectiveness of low power NIR vs. high power NIR for antibacterial applications.

The primary reasons for the translation of photothermal therapies (PTT) to the clinics are the high energy demands for bacterial ablation and the resultant physiological and logistical barriers. By focusing on the development of low-power near-infrared (NIR) solutions, especially those utilizing the NIR-I or II bio window or synergistic treatment modalities, the researchers have started to address these limitations (Zhang et al., 2019; Liu et al., 2021). The use of a high-power (e.g., ≥1 W/cm^2^) NIR source on the bacterial biofilms at the infectious wound site generates heat higher than normal temperatures, which may result in injury of adjacent healthy tissues (Zhang et al., 2019), as in Figure 1. The thermal impact may injure native tissues and result in secondary injuries (Huang et al., 2019). It can also cause collateral thermal burns in surrounding tissues, delaying the healing of the wound. The American National Standard for Safe Use of Lasers (ANSI Z136.1-2014) has determined that for prolonged human skin exposure to an 808 nm laser, the maximum laser power density should not exceed 0.33 W/cm^2^ (Zhang et al., 2022). As a result, in a clinical setting, the potential for thermal injury from conventional PTT systems that function above this threshold is unacceptable (Zhao et al., 2020). However, it is not asserted that anything below 1 W/cm^2^ is safe. At 808 nm (NIR I), the skin highly absorbs and scatters light where power densities greater than 1 W/cm^2^ generate a lot of heat on the top layer of the skin. Therefore, it becomes skin-compliant only when the power intensity is less than 0.33 W/cm^2^. At 1064 nm (NIR II), the light penetrates a larger volume of tissue with less heat. Thus, a clinical implementation requires a thorough assessment of the need for a point source or a distributed source with power densities and durations adjusted accordingly with respect to the infection.

## 3 Low-power NIR solutions as an emerging frontier in the phototherapy field

As a new frontier, the field is rapidly adapting materials with high-efficiency photothermal and photodynamic capabilities or synergistic modalities to use low-power NIR due to limitations of high-power use (Wang et al., 2022; Zhang et al., 2022). By effectively destroying bacteria at lower power densities, this method protects the surrounding healthy tissues (Polakova et al., 2025). Photosensitizers (PS) are the materials that excite with an NIR source and generate heat and ROS helping with PDT and PTT. Table 1 provides a list of PS along with different composites that work under low-power NIR.

**TABLE 1:** Near-infrared (NIR) activated photosensitizers including ≥1 W/cm^2^, their photothermal efficiency and target pathogens.

Sl.no	Photosensitizers	NIR light wavelength and power	Photothermal efficiency/time of irradiation	Target pathogen	References
1	AIE based material (aggregation-induced emission nanoparticles)	808 nm, 0.2 W/cm^2^	5 min	*S. aureus*	Wang et al., 2022
2	Red phosphorous and pure grade Ti plates	808 nm, 0.5 W/cm^2^	10 min	*S. aureus*	Huang et al., 2019
3	PTDBD (a positively charged conjugated polymer)	808 nm, 1 W/cm^2^	31%, 8 min	*S. aureus*, *E. coli*, *C. albicans*	Zhou S. et al., 2020
4	TG-NO-B (TG-thiolated graphene, NO- nitric oxide, B- boronic acid)	808 nm, 0.75 W/cm^2^	37.6%, 10 min	*A. baumanii*, *K. pneumoniae*, *P. aeruginosa*	Zhao et al., 2020
5	Oleate-capped LiYF_4_: Yb/Er UCNPs (up conversion nanoparticles)	980 nm, 0.5 W/cm^2^	12.5 min	*S. aureus*, *E. coli*	Zhang et al., 2018
6	Gold nano stars embedded PDMS films	808 nm, 0.246 W/cm^2^	30 min	*S. aureus*, *E. coli*	Toci et al., 2021
7	Mesoporous polydopamine nanospheres	808 nm, 1 W/cm^2^	28.9%, 6 min	*S. aureus*	Hou et al., 2022
8	SCM@HA nanocomplex (CuS deposited amino functionalized mesoporous silica modified with sodium nitroprusside and hyaluronic acid)	808 nm, 1 W/cm^2^	35.66%, 10 min	*S. aureus*, *S. typhimurium*	Yang J. et al., 2024
9	MoS2@PDA-PEG/IgG	785 nm, 0.58 W/cm^2^	10 min	*S. aureus*	Zhang et al., 2019
10	Nano-TiO_2_ coated on titanium alloy (Ti6Al4V@TiO_2_)	808 nm, 0.8 W/cm^2^	15 min	*S. aureus*, *E. coli*	Wang et al., 2025
11	Rough carbon-iron oxide nanohybrids	1064 nm, 0.5 W/cm^2^	5 min	*S. aureus*, *MRSA*, *E. coli*	Liu et al., 2021
12	Au-Cu Janus nanostructures	1064 nm, 1 W/cm^2^	42.14%, 10 min	*S. aureus*, *E. coli*	Yang Q. et al., 2024
13	Poly (selenoviologen)-assembled up conversion nanoparticles	980 nm, 0.150 W/cm^2^	52.5%, 5 min	*MRSA*	Zhou K. et al., 2020
14	Plasmonic molybdenum oxide nanosheets supported silver nanocubes	808 nm, 0.5 W/cm^2^	10 min	*S. aureus*, *E. coli*	Yin et al., 2018
15	Au nanostars@PDA-ICG	808 nm, 0.75 W/cm^2^	6 min	*MRSA*, *P. aeruginosa*	Yao et al., 2025
16	Sericin-coated gold nanorods	850 nm + 940 nm, 0.391 W/cm^2^	10 min	*S. aureus*, *E. coli*	Poomrattanangoon et al., 2024
17	Plasmonic TiN nanobars and nanosphere	940 nm, 0.318 W/cm^2^	20.2%, 18.1%, 10 min	*S. aureus*	Polakova et al., 2025
18	CuS nanoparticle	900 nm, 0.17 W/cm^2^	8 min	*S. aureus*, *E. coli*	Gulin-Sarfraz et al., 2024
19	Unzipped CNT/PDA (carbon nanotubes and mussel-inspired polydopamine)	808 nm, 0.5 W/cm^2^	10 min	*B. subtilis* and *E. coli*	Patil et al., 2023
20	AuAg yolk-shell cubic nanoframes	808 nm, 0.27 W/cm^2^	65.6%, 10 min	*P. aeruginosa*, *K. pneumoniae*, *B. bacillus*, *E. coli*, *S. aureus*	Zhang et al., 2022
21	TiO_2_:FYH/Cur/BMP-2 (up conversion elements doped titanium dioxide nanorods (TiO_2_ NRs)/curcumin/hyaluronic acid/bone morphogenetic protein2)	1060 nm, 0.6 W/cm^2^	15 min	*S. aureus*, *E. coli*	Zhang et al., 2021
22	UCNPs, e.g., NaYF4:Yb,Er/Tm	980 nm, 0.150 W/cm^2^	52.5%, 4 min	*MRSA*	Zhou K. et al., 2020
23	Au@Ag@SiO2 nanorods	785 nm, 0.50 W/cm^2^	20 min	*E. coli*	Hu et al., 2015
24	AMP–π-extended porphyrin conjugate	720 nm, 0.40 W/cm^2^	15 min	*E. coli*	Gourlot et al., 2022
25	Bimetallic nanozymes	980 nm, 0.6 W/cm^2^	43.18%, 41.23%, 10 min	*E. coli*, *P. aeruginosa* and *S. aureus*	Li et al., 2025
26	Poly (vinylidene) fluoride (PVDF) based nanocomposite membrane	808 nm, 0.5 W/cm^2^	10 min	*S. aureus*, *E. coli*	Cui et al., 2023
27	PDANSs (polydopamine nanospheres)	808 nm, 0.5 W/cm^2^	180 s	*S. aureus*	Ye et al., 2020
28	IND-Cy7(Py)-TCF (indole heptamethine cyanine pyridine)	808 nm, 0.3 W/cm^2^	24.2%, 10 min	*MRSA* and *E. coli*	Hao et al., 2024
29	OGF hydrogel (oxidized dextran gallic acid grafted gelatin and ferric ion)	808 nm, 0.8 W/cm^2^	14 min	*E. coli*, *S. aureus*	He et al., 2023
30	Chiral gold nano-bipyramids	808 nm, 0.8 W/cm^2^	10 min	*S. aureus*	Chen et al., 2022
31	PEG-MoS_2_ nanoframes	808 nm, 1–0.5 W/cm^2^	10 min, 43.72%	*E. coli*	Yin et al., 2016
32	Glycoconjugates capped gold nano rods	808 nm, 0.2 W/cm^2^	5 min	*E. coli*, *P. aeruginosa*	Kaushal et al., 2019
33	Van-CuInSe_2_/ZnS QDs	808 nm, 0.7 W/cm^2^	10 min	*S. aureus*	Geng et al., 2025
34	AP/PT self-assembled NIR-II nanoplatform (amphiphilic polymer and photothermal polymer)	808 nm and 1064 nm, 1 W/cm^2^	10 min	MRSA	Wu et al., 2025
35	Ag_2_S dispersed g-C_3_N_4_ sheets	808 nm, 0.256 W/cm^2^	30 min	*S. aureus*, MRSA	Panda and Neerugatti, 2026

Antibacterial effects can be improved by combining PTT with additional modalities such as PDT or Chemodynamic Therapy (CDT), allowing for energy or dosage reduction for each modality (Liu et al., 2021). Effective killing can occur at lower photothermal temperatures if PDT/CDT’s reactive oxygen species (ROS) increase the material’s permeability and sensitivity to heat. For example, one type of synergistic chemo-PTT platform (TG-NO-B) completely killed bacteria using only a moderate NIR laser density (0.75 W cm^–2^) and attained an equilibrium temperature of 49.8 °C (Zhao et al., 2020). New agents that are efficient and regulatory-compliant are currently in development. Yolk-shell nano frames (AuAg YSCNFs) achieved the highest photothermal conversion efficiency (65.6%) with an NIR laser at a very low power density of 0.27 W cm^–2^ (below the skin-tolerance limit) (Zhang et al., 2022). UCNPs/PSeV, an additional hybrid photosensitizer, showed synergy under the lightest irradiation conditions reported so far (λ = 980 nm, 150 mW/cm^2^, 4 min) (Zhou K. et al., 2020). An important part of this trend is the development of agents that target the NIR-II bio-window (1000–1700 nm), which is beyond the established NIR-I window (650–950 nm) (Liu et al., 2021). NIR-II PTT systems contain various advantages such as deeper tissue penetration, less energy dissipation, higher skin tolerance, and low toxicity (Zhang et al., 2021). For example, rough carbon-iron oxide nanohybrids (RCF) have low power density (0.5 W/cm^2^) and improved penetration in the NIR-II window, enabling synergistic PTT/CDT (Liu et al., 2021). Furthermore, studies in animal models revealed that NIR-II light (1060 nm, 1 W/cm^–2^) penetrated deeper into tissues than 808 nm light and correspondingly produced a greater increase in temperature at depth (Zhang et al., 2021).

The research focuses on developing stable, efficient systems that are triggered by low-power single light sources. Organic photothermal agents like IND-Cy7(Py)-TCF possess high photothermal conversion efficiency and singlet oxygen yield (ΦΔ) upon irradiation with single-wavelength NIR excitation over 800 nm (Hao et al., 2024). Cationic chalcogenoviologen derivatives are also applied in photodynamic antimicrobial therapy (Zhou K. et al., 2020). Multifunctional systems combine various treatment modalities to achieve combined antimicrobial effects and enhance efficacy (Liu et al., 2021).

Photothermal-Photodynamic/Chemodynamic (PTT-PDT/CDT) is a common synergistic modality (Wang et al., 2022). For example, Aunst@PDA-ICG (gold nanostars coated with Indocyanine Green) generates both thermal energy (∼53.2 °C) and ROS upon near-infrared (NIR) irradiation (Yao et al., 2025). UCNPs/PSeV facilitates PTT and PDT use with a single near-infrared (NIR) light source (Zhou K. et al., 2020). The AuAg yolk-shell cubic nanoframes combine photothermal therapy (PTT) with the natural antibacterial properties of silver (Ag) (Zhang et al., 2022). Nanohybrids like RCF exploit the peroxidase-like activity of iron oxide (Fe_3_O_4_) for CDT (producing hydroxyl radicals, OH⋅, from H_2_O_2_) and subsequent PTT hypersensitization of bacterial membrane permeability (Liu et al., 2021). Systems are increasingly fusing phototherapy with various bioactive capabilities. For example, NIR laser-activated platforms containing copper sulfide and sodium nitroprusside can achieve synergistic PTT, CDT, and Nitric Oxide (NO) gas treatment, where PTT enhances CDT efficacy (Yang J. et al., 2024). Complex core-shell-shell structures such as Au-Ag-Au nanorods combine the external Au shell’s photothermal therapy with the precisely regulated release of the internal Ag shell/Ag^+^ during NIR irradiation, leading to enhanced stability and antimicrobial efficacy (Hu et al., 2015). Gold nanostructures developed for targeted delivery, loaded with antibiotics, are used for combined photothermal and antibiotic eradication (Zhang et al., 2019). Ultimately, the movement toward producing efficient photosensitizers and combination therapeutic strategies along with NIR based PTT and PDT opens a technological pathway for the future where we can witness safe, portable and accessible photothermal applications in clinics (Poomrattanangoon et al., 2024).

## 4 Discussion

The use of NIR active photosensitizers for antimicrobial applications offers progressive non-antibiotic strategies that aim at enhancing effectiveness and adaptability for clinical use, having no fear for resistance development (Hao et al., 2024). Phototherapeutic agents summarized in Table 1 reveal several trends regarding material design and therapeutic efficacy. Structurally, the field is undergoing a clear transition from traditional inorganic nanomaterials such as gold nanostructures and MoS_2_ nanosheets to highly engineered, multifunctional organic and hybrid platforms, including metal-organic frameworks (MOFs), upconversion nanoparticles (UCNPs), and self-assembled amphiphilic polymers. Comparatively, the data highlights that materials relying solely on photothermal therapy (PTT) often require operating power densities closer to the 1.0 W/cm^2^ threshold to effectively eradicate multidrug-resistant pathogens like MRSA. In contrast, nanoplatforms engineered for synergistic modalities, such as combining PTT with photodynamic therapy (PDT), chemodynamic therapy (CDT), or nitric oxide (NO) release, demonstrate a distinct advantage. These synergistic materials consistently achieve high antimicrobial and anti-biofilm efficacy at significantly lower power densities, often ≤0.5 W/cm^2^. Furthermore, the data in Table 1 illustrates a strategic wavelength shift. While many established platforms operate in the NIR-I window (e.g., 808 nm), recent advanced materials (such as the AP/PT polymeric nanoplatforms) are designed for the NIR-II window (e.g., 1064 nm). This shift not only improves deep-tissue penetration but also capitalizes on the higher maximum permissible exposure limits of NIR-II light, ultimately providing a safer, low-power alternative for treating deep-seated biofilm infections without compromising adjacent healthy tissue. However, there is still research that exists where antibiotics are combined with different NIR active materials for antimicrobial applications. It is understood that they may have synergistic effects, but again they are not contributing to antibiotic-free goals. For example, research shows that combining photothermal therapy and photodynamic therapy with drug delivery methods like incorporating levofloxacin into GNRs@MSN (Mesoporous Silica & Gold Nanorods) results in a synergistic antibacterial effect through targeted antibiotic-loaded nanostructures (Zhang et al., 2019). Alternatively, another study used NIR-triggered heat-sensitive liposomes with antibiotics as a combined treatment approach (Hou et al., 2022). These methods of administering the antibiotics seem to be novel and outsmart the conventional way of administration in terms of bioavailability. An enhanced as well as targeted delivery approach involves fabricating materials that enable controlled and prolonged drug release, activated by near-infrared light. For example, integration of photothermal agents along with therapeutic drugs into a polymer base can be utilized for PTT and PDT where sustained release of drugs such as curcumin can be expected with an NIR trigger (Patil et al., 2023). Surface disinfection has become a primary focus in some current research where eradication of either planktonic bacteria or their biofilms on medical devices and catheters is performed via NIR based PTT or PDT (Toci et al., 2021). For example, NIR-II activated photosensitizers fabricated using TiO_2_ nanorods, curcumin (QSIs), and BMP-2 for titanium implants to effectively remove biofilms at moderate temperatures (45 °C) and helped in bone integration (Zhang et al., 2021). Low-power NIR active materials are incorporated into conventional biopolymers such as polydimethylsiloxane, investigated for antibacterial applications on the subcutaneous surfaces where prosthetic devices and catheters are inserted (Toci et al., 2021). Concentrating the photothermal agent at the site of infection or biofilm formation by using a bacteria-targeting component is found to be an advanced approach (Zhang et al., 2019). For example, photothermal agents having cationic groups on their surface can be engineered to target bacteria through strong electrostatic interactions with the negatively charged bacterial membrane, allowing for targeted treatment. Furthermore, surface modification like using rough carbon-iron oxide nanohybrids enhances bacterial attachment, leading to more efficient treatment (Liu et al., 2021). Another approach involves utilizing unusual photophysical phenomena such as Anti-Stokes Luminescence which arises from hot band absorption, to enhance deep-tissue penetration and achieve imaging with low background noise (Hao et al., 2024). Combining therapy with diagnostic capabilities improves ease of use and accuracy. For example, titanium nitride nanocrystals serve both as a photothermal therapy agent and as a contrast agent for photoacoustic imaging, enabling visualization of the treatment site and tracking of the therapy’s effectiveness (Polakova et al., 2025).

The success of translation to the real world of phototherapies is constrained by various factors, like complexity in the process, expenses of materials used, and regulatory strategies. Complex multifunctional platforms used in various research for PTT and PDT may face challenges in clinical application because of their poor reproducibility and complex manufacturing processes, which may not meet industrial standards. Therefore, there is a significant demand for materials that are straightforward and easy to produce (Hao et al., 2024; Poomrattanangoon et al., 2024). The expensive price and scarcity of noble metals such as gold and silver continue to limit their widespread use for these therapies (Yin et al., 2018). Affordable options are being explored, including transition metals, which are cheap compared to noble metals and exhibit excellent stability (Polakova et al., 2025). In addition, Polydopamine, commonly utilized for surface modification, is recognized for being affordable and easy to synthesize under mild conditions (Hou et al., 2022) for NIR-based therapies. Utilizing agents with established clinical applications can speed up regulatory approval. For instance, Indocyanine Green, an NIR imaging agent approved by the US FDA is commonly used in PTT/PDT nanosystems even though it has a short half-life and limited effectiveness when used alone (Hao et al., 2024). Materials used for PTT and PDT are required to remain stable when exposed to physiological conditions and heat. For example, Au-Ag-Au nanorods designed with a core-shell structure offer better stability. Mechanistically, this design enables the outer gold shell to melt under low-power NIR laser (785 nm, 50 mW/cm^2^) exposure, acting as a nanoheater, which then initiates the controlled release of the antibacterial Ag components (Hu et al., 2015). Titanium nitride nanospheres exhibit outstanding thermal stability up to 1400 °C and maintain consistent photothermal performance over several light-on or light-off cycles, highlighting their promise as a long-lasting photothermal therapy agent (Poomrattanangoon et al., 2024). The effective use of multifunctional systems in animal models for wound healing offers a solid foundation for future clinical applications, especially in treating chronic issues such as diabetic wounds (Yin et al., 2016). Progress in creating nanozymes such as PdCu/PdZn has led to a design approach that can be used not only for clinical wound care but also for antimicrobial surface coatings and ensuring food safety (Li et al., 2025). The primary drawbacks of nanomaterials are their microscopic size and high surface reactivity, which sometimes bypass natural biological defenses, infiltrate cells, and induce severe oxidative stress or DNA damage (Oberdörster et al., 2005). Furthermore, because their toxicity is heavily dependent on highly variable physical factors like shape, size, and surface charge rather than just chemical composition, standard toxicological assays often yield unpredictable results, making standardization of safety regulations difficult (Nel et al., 2006; Moore, 2006).

## 5 Future direction and perspectives

There is a great bridge needed in translating the above-discussed therapeutic agents to real-world applications. Only a few clinical trials focus on infectious disease treatment using NIR. For example, the photo-disinfection using chitosan nanoparticles trial (NCT06523244) has been investigated with nanoparticle-enhanced indocyanine green photodegradation, which helps in disrupting bacterial biofilms and helps with immunomodulation in periodontal disease. The dye is activated with an 810 nm diode laser with continuous wave frequency and power of 0.3 W with a 400 μm fiber tip for 20 s/spot. In dentistry, a clinical trial being conducted examines how laser-activated nanoparticles can efficiently remove stains without affecting the enamel integrity (NCT02353611). During the treatment, a gel’s surface was activated by continuous light irradiation for 12 min using LED/laser light with a total power of 1800 mW (Chauhan et al., 2026). Periodontal Disease (Adjunctive antimicrobial PDT) registered RCT (NCT05962801) evaluated daily home-applied dual-light PDT as an aid to non-surgical periodontal therapy (NSPT) in smokers with Stage III/IV periodontitis over 6 months (January 2023–June 2024), demonstrating significant improvements in bleeding on probing, probing depth and clinical attachment levels (Vakaki et al., 2025). Meanwhile, an antimicrobial PDT for periodontal disease in diabetic patients involved a clinical trial (NCT05816941) that assessed ICG-mediated antimicrobial PDT as an adjunct therapy in patients with Type 2 diabetes mellitus and periodontitis, showing significant reduction in BoP (bleeding on probing) and lower presence of periodontal pathogens like *T. forsythia* in the PDT group compared to controls. For this purpose, a Fotona XD-2 diode laser (Fotona, Ljubljana, Slovenia) with a wavelength of 810 nm, a power of 250 mW, and the photosensitizing agent indocyanine green at a concentration of 1 mg/ml was used (Brinar et al., 2023). Phototherapies like PTT and PDT use nanoparticles, which need to face a complicated regulatory approval compared to any other pharmaceuticals due to their hybrid character and unique modes of action (Zhang et al., 2024). However, there are some phototherapeutic drugs approved by the FDA that stand out to showcase the transformative potential of such therapies, especially for the treatment of cancer. For example, Photofrin^®^ and Visudyne^®^ have gained FDA approval (Leunig et al., 1994; Baskaran et al., 2018). It is clear from the above discussion that regulatory approval requires thorough validation of physicochemical characteristics, repeatability, and long-term safety evidence. Furthermore, there are not any consistent criteria for phototherapeutic nanomedicines from global regulatory authorities like the US Food and Drug Administration and the European Medicines Agency, which complicates the clearance process. Furthermore, early-phase clinical trials are few, and hence, larger multicentric studies are required to prove efficacy, safety, and optimal dose regimes (Rastinehad et al., 2019). To bridge the translational gap, phototherapy devices must be integrated into existing clinical processes, which will be backed by evidence-based guidelines and real-world examples.

Despite major breakthroughs, there is still a lot of room for improvement. When using low-power NIR, phototherapy can specifically target infection sites while limiting injury to healthy cells in the vicinity, which remains one of its most attractive features. Particularly, PDT-induced ROS production disturbs homeostasis in cells, resulting in death, whereas PTT-induced hyperthermia completely destroys microbial cellular structures and activates stress response pathways that enhance immunological responses at the infection site. Furthermore, the ability to cause immunogenic bacterial ablation has two benefits: it eliminates infections while simultaneously inducing systemic immunity to infections (Chauhan et al., 2026).

Meanwhile, the total fluence (Power Density × Time) can be introduced as a standard for the materials rather than denoting just the power density of a NIR active material to establish a quick analysis of their efficiency and safety concerns. In addition, the dark toxicity (ROS generation) of nanomaterials must be strictly evaluated while analyzing the light-induced heating limit. The accumulation of nanomaterials in the system may outweigh their benefits for NIR therapy. Also, the current clinical trials predominantly use FDA-approved dyes like indocyanine green. However, there exists a huge gap in understanding how a complex nanomaterial would perform under a clinical trial. Future research should focus on finding new molecular hits and roads to improve therapeutic outcomes. For example, targeting hypoxia-inducible factors (HIFs) in the infectious microenvironment could improve the efficacy of PDT and PTT in hypoxic conditions. Similarly, safe-by-design approaches would enhance the applicability of the nanomaterials, reducing their concern regarding toxicity. Furthermore, the development of multifunctional nanoparticles having both diagnostic and therapeutic properties with NIR activation will allow for real-time monitoring of treatment efficacy. Studies on NIR-II-responsive nanomaterials are also promising, since these have the potential to answer the current limitation of light penetration, allowing for deeper tissue treatment and broadening the variety of infections that can be efficiently targeted.

## 6 Conclusion

In addition to advancements, more studies are required for biocompatibility, biodegradability, and large-scale production, which will always be a critical aspect to bring phototherapy into mainstream clinical use. Innovative eco-friendly synthesis methods and affordable fabrication processes are critical in assuring accessibility and affordability for a global patient base. Conclusively, the combination of advanced nanomaterials and their mechanism of action at the molecular level can bring deeper understanding of PTT and PDT for infectious disease. Most of the studies we reviewed focus solely on superficial infections. The studies should be broadened to treat visceral infections for which advanced targeted delivery mechanisms and the fate of photosensitizers are to be decided. However, there are major issues still present for the translation of such phototherapies to a real-world, standardized regulatory framework for phototherapeutic nanomedicines from concerned agencies, poor reproducibility and complex manufacturing, absence of large multicentric clinical trials for antimicrobial phototherapy, and finally, Unknown long-term nanotoxicity and biodistribution in humans. Progressive research and innovation will be essential to realize its potential for translation to healthcare.
